# Effects of Omega-3 Polyunsaturated Fatty Acids Intake on Vasomotor Symptoms, Sleep Quality and Depression in Postmenopausal Women: A Systematic Review

**DOI:** 10.3390/nu15194231

**Published:** 2023-09-30

**Authors:** Ayesha Zafar Iqbal, Suet-Kei Wu, Halliru Zailani, Wei-Che Chiu, Wen-Chun Liu, Kuan-Pin Su, Shin-Da Lee

**Affiliations:** 1 Graduate Institute of Nutrition, China Medical University, Taichung 404, Taiwan; ayeshamir146@gmail.com (A.Z.I.); wskei23@gmail.com (S.-K.W.); halliruzyln55@gmail.com (H.Z.); 2 Mind-Body Interface Research Center (MBI-Lab), China Medical University Hospital, Taichung 404, Taiwan; 3 Department of Psychiatry, Cathay General Hospital, Taipei 106, Taiwan; doctor.chiu@gmail.com; 4 School of Medicine, Fu Jen Catholic University, Taipei 242, Taiwan; 5 An-Nan Hospital, China Medical University, Tainan 709, Taiwan; graceliu8911@gmail.com; 6 College of Medicine, China Medical University, Taichung 404, Taiwan; 7 Ph.D. Program in Healthcare Science, Department of Physical Therapy, China Medical University, Taichung 404, Taiwan

**Keywords:** amenorrhea, depression, hot flashes, omega-3 PUFA, post-menopause, sleep quality, vasomotor symptoms

## Abstract

The menopausal transition is often accompanied with distressing manifestations, such as vasomotor symptoms, sleep disruptions, and depressive syndrome. Omega-3 polyunsaturated fatty acids (*n*-3 PUFAs) have emerged as a potential intervention to alleviate these symptoms. This review aimed to comprehensively assess the impact of *n*-3 PUFAs supplementation on vasomotor symptoms, sleep quality, and depression among postmenopausal women. We conducted a systematic literature search of randomized controlled trials across the Cochrane Library, Web of Science, PubMed, CINAHL, EMBASE, and SCOPUS databases from inception to August 2023. Among the initial pool of 163 identified studies, nine studies met the inclusion criteria and were incorporated into this systematic review. Notably, four studies detected potential benefits of *n*-3 PUFAs in improving hot flashes and night sweats. On the contrary, sleep quality outcomes displayed heterogeneity across the studies. Incorporating diverse scales, such as the Hamilton Depression Rating Scale-21, the Patient Health Questionnaire depression scale, and Generalized Anxiety Disorder-7 for depression outcomes, we found inconclusive evidence of *n*-3 PUFA’s impact on depression. Overall, the combined analysis of these studies did not provide substantial evidence to support the efficacy of *n*-3 PUFAs in improving vasomotor symptoms, sleep quality, and depression. Further well-designed randomized clinical trials with larger participant groups are crucial to validate and generalize these results. Review Registration: PROSPERO registration no: CRD42023421922.

## 1. Introduction

Menopause, a phase marked by complex physiological changes in women, significantly impacts their well-being [1]. Defined as a cessation of menstruation for approximately one year after the last menstrual cycle, menopause spans 40 to 60 years, with an average age of 52 [2]. Vasomotor symptoms (VMS), encompassing hot flashes (HF), and night sweats, alongside various other manifestations such as sleep disturbances, anxiety, depression, vaginal dryness, muscular discomfort, and sexual dysfunction, collectively impair the quality of life during this period [3,4]. It has been discovered that 30–44% of women who experience moderate to severe VMS meet the diagnostic criteria for persistent insomnia. In contrast, among women who do not have VMS, this number is significantly lower, at only 11% [5]. Furthermore, HF and major depressive disorder (MDD) emerge as prominent symptoms, with HF occurring in up to 80% of cases [6] and MDD affecting over 20% [7] of menopausal women. Consequently, more than 1/3rd of women obtain medical assistance due to the discomfort induced by HF [8]. Menopause-related depression needing medication has a substantial age of onset; it is more prevalent (10–15%) when symptoms begin before 45, but less common (5–6%) when symptoms begin at 48 or later [9].

The origins of HF remain not fully elucidated but are theorized to result from disturbances in temperature regulation, possibly linked to factors such as fluctuations in estrogen levels and alterations in neurotransmitter function [10,11]. The decline in estradiol (E2) levels is known to disrupt both the hypothalamic-pituitary-adrenal (HPA) axis and serotonergic systems, which subsequently trigger VMS, including hot flashes and night sweats. These VMS, in turn, disrupt normal sleep patterns through mechanisms such as stress-induced cortisol elevation and serotonin dysregulation. The intricate interplay between hormonal changes, particularly the decrease in estrogen, and disrupted sleep is thought to contribute to the development of depressive symptoms [12,13]. 

Estrogen Therapy (ET) is a common therapy used to treat postmenopausal symptoms and age-related sarcopenia. ET works by increasing myogenic gene expression, myogenic regulatory factors (myogenic differentiation factor (MyoD), myogenic factor 5 (Myf5), muscle regulatory factor 4 (MRF4), and myogenin), and muscle satellite activation and proliferation [14,15]. Hormone therapy (HT) remains the prevailing strategy for mitigating VMS, yet it harbors a spectrum of associated risks and potential adverse outcomes [16]. Integral healthcare for menopausal women should prioritize lifestyle assessment and counseling, with a specific emphasis on nutrition, as it impacts overall well-being and quality of life in the postmenopausal period by mitigating the negative effects of estrogen deficiency [17]. Currently, certain vitamins (A, D, E, and B-complex) and omega-3 polyunsaturated fatty acids (*n*-3 PUFAs) are playing a role in the menopausal transition symptoms, but the evidence to support their definite roles is still inconclusive. 

**Figure 1:**
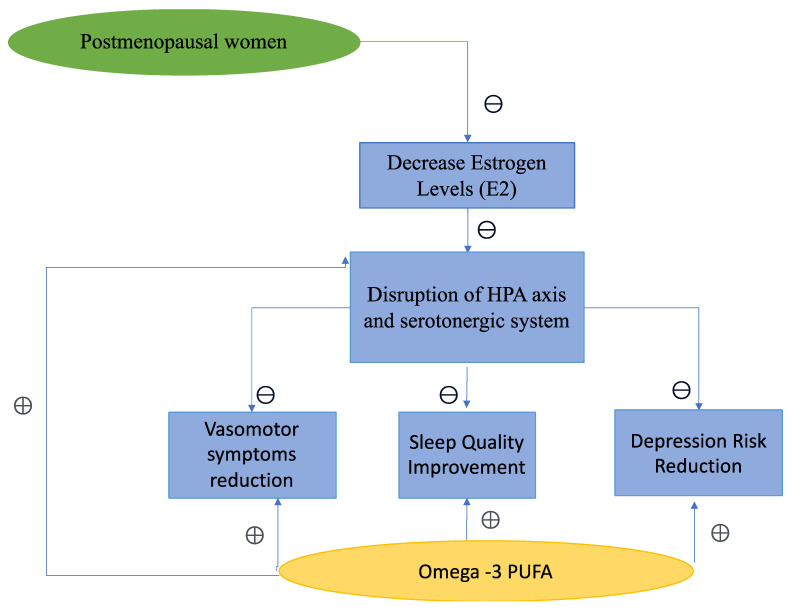
*n*-3 PUFAs’ role in improving vasomotor symptoms sleep quality and depression. Plus (+), and minus (−) signs represent a positive and negative relationship.

*n*-3 PUFAs, including eicosapentaenoic acid (EPA), docosahexaenoic acid (DHA), and alpha-linolenic acid (ALA), represent essential dietary components with multiple double bonds [18]. Renowned for their therapeutic potential, these long-chain *n*-3 PUFAs supplements have been utilized for treating diverse medical conditions such as cardiovascular disease, depression, and cognitive disorders [19,20]. Their efficacy in addressing menopausal symptoms and MDD in perimenopausal and postmenopausal women has also been investigated [21,22,23]. Human and animal investigations elucidating the mechanistic underpinnings of *n*-3 PUFAs indicate their involvement in the regulation of serotonergic and dopaminergic neurotransmitter systems. However, the definitive favorable impact of *n*-3 PUFAs on menopausal transition-associated HF, depression, and cognitive symptoms remains inconclusive [24,25,26] (Figure 1). 

In a clinical investigation, supplementation of ethyl EPA resulted in reduced HF and improved HF scores compared to a placebo [27]. However, another study failed to observe any significant alterations in VMS and sleep quality when compared to a placebo [28]. A comprehensive review of 32 studies underscored the vulnerability of menopausal women to depression and anxiety [29]. Exploring the interplay of HF, sleep patterns, and depression in women undergoing menopause induced by a Gonadotropin-releasing hormone (GnRH) agonist medication, a study revealed significant associations between increased sleep interruptions, nocturnal HF, and heightened depression scores [30]. Although certain studies supported the potential of *n*-3 PUFAs in mitigating depression and HF [31,32], discrepant evidence arises from studies that found no support for the influence of *n*-3 PUFAs on depression scores, as assessed through diverse rating scales [28]. 

Presently, the precise impact of *n*-3 PUFAs supplementation on VMS remains elusive. Convergent research in both animal and human subjects suggests that *n*-3 PUFAs may modulate neurotransmitter levels, including serotonin and dopamine, within the brain by elevating levels of these fatty acids [25,33]. Consequently, the definitive impact of *n*-3 PUFAs supplements on VMS, sleep quality, and depression scores lacks empirical validation. As such, this systematic review endeavors to synthesize existing evidence to elucidate the efficacy of *n*-3 PUFAs supplementation in ameliorating VMS, enhancing sleep quality, and reducing depression scores in the context of postmenopausal women.

## 2. Materials and Methods

### 2.1. Study Search Strategy and Selection

The systematic review was performed according to the systematic reviews and metanalysis (PRISMA) guidelines [34]; details are available in Supplementary Table S1. The study’s registration in the Prospective Register of Systematic Reviews (PROSPERO) was completed under registration number CRD42023421922. The PICOS (Patients, Intervention, Comparison, Outcome, Study Design) paradigm was used to develop the search terms (Table 1). The PICO question was “How do *n*-3 PUFAs affect vasomotor symptoms, sleep quality, and depression in postmenopausal women?”

Multiple databases, including the Cochrane Library, Web of Science, PubMed, Embase, CINAHL, and SCOPUS, were utilized. The search employed both free text and Medical Subject Headings (MeSH) terms, such as “omega-3”, “fish oils”, “PUFA”, “menopause”, “hot flashes”, “night sweats”, “vasomotor”, “sleep quality”, “insomnia”, and “depression”. Supplementary sources, such as Google Scholar and ClinicalTrials.gov, were also consulted to identify ongoing or unpublished research. The search encompassed studies published in English from inception to the present, without imposing restrictions on publication time or status. A comprehensive search strategy was executed to identify pertinent studies for this systematic review presented in the Supplementary Table S2.

### 2.2. Included and Excluded Studies

The systematic review applied the following inclusion criteria: (1) Randomized controlled trials (RCTs) featuring a single intervention group receiving *n*-3 PUFAs supplementation in comparison to a placebo or alternative control group; (2) Encompassing studies involving both naturally postmenopausal women (defined as having experienced more than 12 months since their last amenorrhea) and surgically postmenopausal women (as indicated by follicle-stimulating hormone levels exceeding 40 IU/L); (3) Inclusion of studies regardless of the administered *n*-3 PUFAs dosage; (4) Studies that reported relevant outcomes pertaining to postmenopausal symptoms, including but not limited to hot flashes, night sweats, mood disturbances, sleep quality, and depression.

Exclusion criteria comprised: (1) Studies lacking the reporting of pertinent outcomes or those without access to full-text publications; (2) Observational and non-human studies; (3) Studies published in languages other than English.

### 2.3. Study Participants

The study focused on women at both menopausal and post-menopausal stages, who were experiencing VMS and depression due to menopause, or women undergoing surgical menopause who were also experiencing VMS and depression.

### 2.4. Type of Intervention and Control

Included studies evaluated *n*-3 PUFAs supplementation at any dosage, frequency, and form (capsule, oil, powder) compared to placebo or other control groups. Studies involving fish consumption, use of antidepressants, hormone replacement therapy, use of anticoagulants, and those lacking placebo or adequate control groups, were excluded.

### 2.5. Outcome Measures

The primary outcomes targeted VMS, including the frequency and intensity of HF and night sweats, which were assessed through patient-maintained diaries or measured using scales such as the Hot Flash-Related Daily Interference Score Kupermann index, and menopause rating scale. Other primary outcomes included sleep quality and depression, measured using established scales including the Pittsburgh Sleep Quality Index, Insomnia Severity Index, Beck’s Depression Inventory, Montgomery–Asberg Depression Rating Scale, Generalized Anxiety Disorder Questionnaire, 20-item Hopkins Symptom Checklist Depression Scale, 21-item Hamilton Depression Rating Scale, and Physician’s Health Questionnaire depression domains. Secondary outcomes encompass menopause-specific quality of life scores and the monitoring of adverse events.

### 2.6. Data Extraction and Quality Assessment

Three reviewers (A.Z., S.K., and S.D.L.) screened the titles and abstracts in stage one of the screening. A third member (K.P.S.) resolved disagreements. In stage two, full papers extracted from the previous stage were independently screened by two reviewers (A.Z., and S.K.). To begin, nine papers were chosen to determine reviewer consistency. The quality of the included studies was assessed using the Cochrane Collaboration Risk of Bias Assessment Tool by two reviewers (A.Z. and S.K.) separately [35]. The third member (K.P.S) resolved any apparent discrepancy resulting from the assessment process. To assess the quality of studies, Cochrane’s seven areas of assessment were used: randomization, allocation concealment, blinding of participants, blinding of outcome assessment, inadequate outcome data, selective reporting, and additional bias.

### 2.7. Statistical Analysis

The initial plan was to perform a meta-analysis to quantify the overall effect of *n*-3 PUFAs supplementation on VMS reduction, sleep quality improvement, and depression risk reduction as the outcome measures with respect to the control or placebo group. However, the inability to conduct a quantitative synthesis in this study is attributed to the substantial heterogeneity observed among the included studies for several reasons. First, variations in the dosages and frequencies of *n*-3 PUFAs supplementation were prevalent across these studies, making it challenging to combine their findings in a statistically meaningful way. Secondly, the utilization of diverse assessment methods for measuring outcomes introduced a potential source of bias and measurement error, further complicating the comparability of results between studies.

## 3. Results

### 3.1. Selected Studies

**Figure 2:**
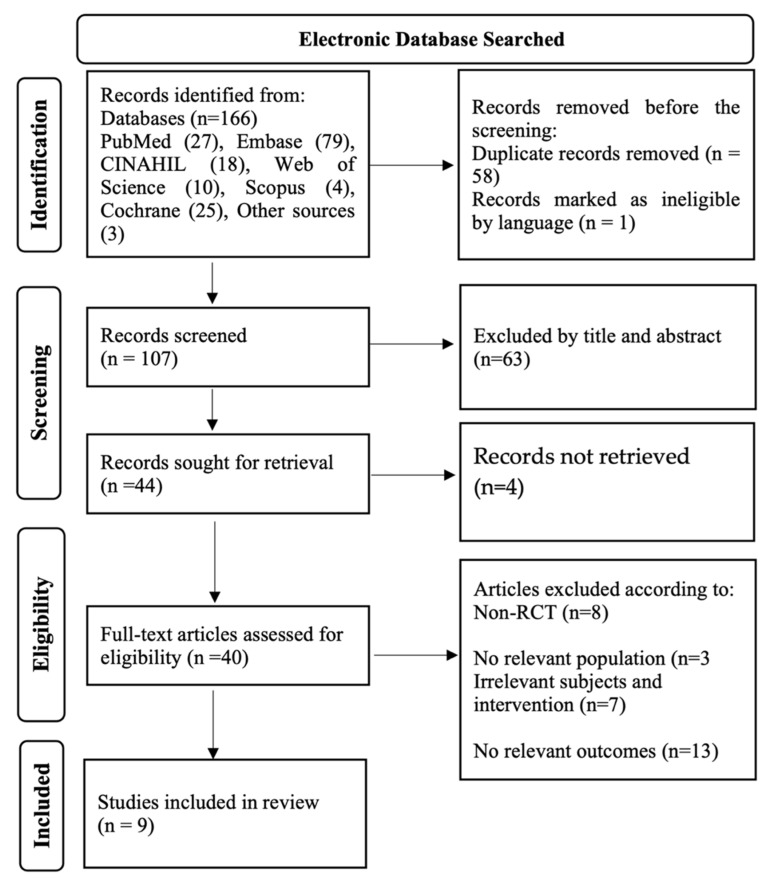
PRISMA flowchart for study search and selection strategy.

Figure 2 depicts the results of the screening process. The database searches yielded a total of 163 studies; after eliminating 58 duplicate entries, 107 publications were reviewed. After selection based on the title and abstract, 44 publications were selected. Further, information regarding other excluded articles can be found in Supplementary Materials Table S3. Finally, this systematic review identified nine relevant papers that contained RCTs with sample sizes ranging from 60 to 546 people. These studies evaluated how *n*-3 PUFAs supplements affect menopausal symptoms, sleep quality, and depression in menopausal women. The participants in the RCTs were given varying amounts of EPA and DHA, the *n*-3 PUFAs of interest. Depending on the trial design, the placebo groups received soybean oil, sunflower oil, or olive oil. Furthermore, some research used interventions other than the aforementioned placebos, extending the types of interventions used.

### 3.2. The Effect of n-3 PUFAs on VMS

The studies evaluating the impact of *n*-3 PUFAs supplementation on menopausal symptoms are summarized in Table 2. Two RCTs detected no significant difference in the frequency of vasomotor symptoms (VMS) with *n*-3 PUFAs supplementation [28,36], whereas others found a decrease in both VMS and HF frequency and intensity [27,37,38]. Moreover, a separate study found a decreased HF frequency but no effect on the intensity [39]. Overall, this systematic review indicates that *n*-3 PUFAs supplementation may have a variable impact on menopausal symptoms, with some studies showing a decrease in symptoms and others reporting no significant changes.

### 3.3. The Effect of n-3 PUFAs on Sleep Quality

Three studies examined the effect of *n*-3 PUFAs on the sleep quality of postmenopausal women. Sleep quality was evaluated using the PSQI and ISI measures in all the studies. Specifically, Reed et al. found that *n*-3 PUFA supplementation had no influence on sleep quality among 355 menopausal women compared to the placebo group [36]. Similar to this, a double-blind, randomized clinical trial by Cohen et al. found no effect in menopausal women taking 615 mg of *n*-3 PUFAs daily for 12 weeks [28]. Moreover, an increased daily intake of 1.8 g of *n*-3 PUFAs also had no impact on sleep quality, according to Guthrie et al. In the study, *n*-3 PUFA supplements did not appear to have a substantial impact on postmenopausal women’s sleep quality compared with placebo [41]. Based on the existing research, this systematic review concludes that *n*-3 PUFA supplementation does not appear to have a substantial impact on sleep quality in postmenopausal women.

### 3.4. The Effect of n-3 PUFAs on Depression

This systematic review included four studies investigating the effects of *n*-3 PUFA supplementation on depression in menopausal women. Masoumi et al.’s triple-blind, randomized controlled trial demonstrated that menopausal women who received a combination of 20 mg citalopram and 1 g of *n*-3 PUFAs showed a decrease in depression as measured by the BDI-II [40]. However, the double-blind, randomized clinical trial conducted by Cohen et al. and Reed et al. observed no statistically significant alterations in depression levels, as evaluated through the PHQ-8 and GAD-7 scales, following a 12-week regimen of 1.8 g/day *n*-3 PUFAs supplementation (425 mg of EPA, 100 mg DHA and 90 mg of other *n*-3 PUFAs, 3 pills/day) [28,36]. In contrast, the double-blind, placebo-controlled study conducted by Lucas et al. revealed a reduction in depression scores (measured using PGWB, HSCL-D-20, and HAM-D-21) among menopausal women administered with 500 mg *n*-3 PUFAs capsules (350 mg EPA and 50 mg DHA) thrice daily over 8 weeks [31]. Overall, our systematic review reveals that *n*-3 PUFA supplementation may improve depressive symptoms in postmenopausal women, as indicated by several of the included studies, even though not all studies showed meaningful changes.

### 3.5. Other Outcomes

Out of the nine studies, the majority of the studies found no adverse effects associated with *n*-3 PUFA supplementation [27,28,37,38,39]. In addition, as indicated by the menopause-specific quality of life score (MENQOL), two studies found an increase in quality of life specifically related to menopause, suggesting a potential positive impact of *n*-3 PUFAs supplementation [27,36]. These findings highlight the safety and potential benefits of omega-3 supplementation for menopausal women.

### 3.6. Risk of Bias Assessment (RoB)

**Figure 3:**
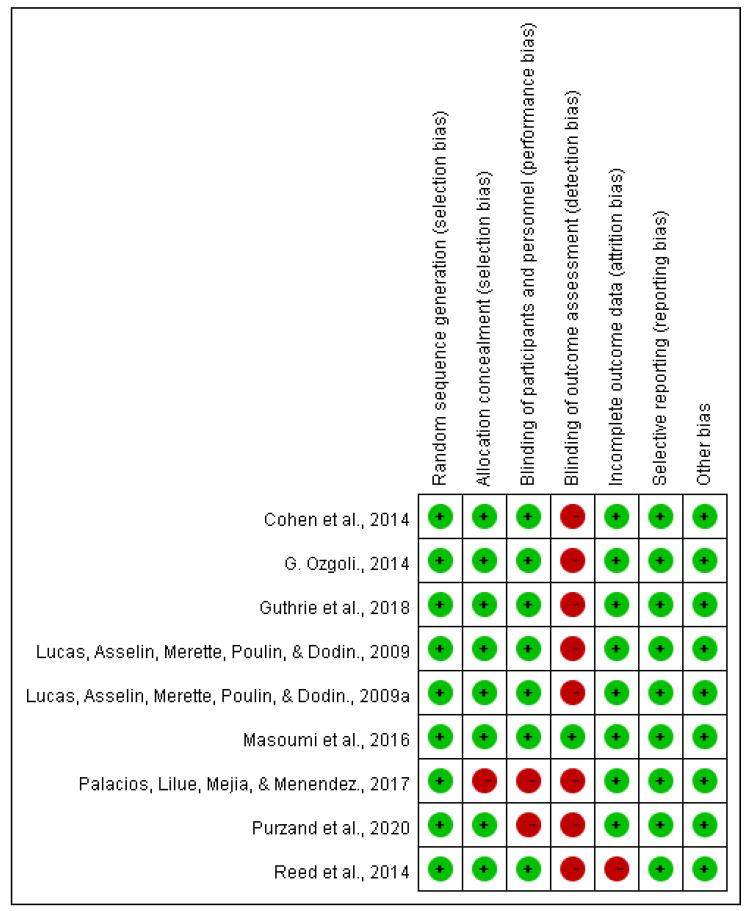
Risk of bias for studies with *n*-3 PUFAs and premenopausal women. Green (+), a low risk of bias; red (−), a high risk of bias. [27,28,31,35,36,37,38,39,40,41].

Figure 3 provides a comprehensive assessment of the methodological quality across the various studies included in our systematic review, employing a diverse set of criteria to gauge potential biases present in their research design and reporting. Each study underwent a meticulous evaluation process, resulting in categorizations of “low”, “high”, or “unclear” to signify the extent of perceived bias. Notably, the analysis uncovers that only a single study manages to demonstrate a low level of bias across multiple domains, encompassing random sequence generation, allocation concealment, blinding of participants, blinding of personnel, management of incomplete outcome data, selective reporting, and other potential sources of bias [40]. However, the majority of the reviewed studies raise concerns, primarily about a high level of bias in the blinding of participants [37,38] and personnel [27,28,31,36,37,38,39,41]. Additionally, there is a somewhat lesser but still noteworthy level of bias observed in the attrition bias [36]. These findings collectively suggest that, while many of the studies exhibit robust methodologies in areas such as randomization and participant blinding, there exists a notable vulnerability to performance and detection biases that could potentially impact the validity and reliability of their reported results. Thus, critical consideration of these biases is imperative when interpreting and drawing conclusions from the body of research examined.

## 4. Discussion

This systematic review aimed to evaluate the impact of *n*-3 PUFAs supplementation on menopausal symptoms in postmenopausal women. The review encompassed nine pertinent randomized controlled trials that exhibited diversity in terms of sample sizes and treatment approaches. The trials were comprehensive in investigating a range of menopause-related issues, including VMS, sleep quality, depression, and various indicators of quality of life.

The menopausal transition signifies a profound period of transformation for women. This natural progression involves a decline in E2 (10–20 pg/mL) levels [42], potentially leading to modifications in brain neurochemicals and instability within the hypothalamus—the brain region responsible for regulating body temperature. These changes are often attributed to the emergence of VMS, encompassing HF and night sweats [43]. HT stands as the foremost efficacious treatment for HFs and holds the sole FDA-approved indication for symptom relief. Nevertheless, a significant number of women currently exhibit reluctance to pursue HT, primarily attributed to apprehensions regarding associated risks [44]. Non-hormonal treatments, such as selective serotonin reuptake inhibitors (SSRIs) or serotonin-norepinephrine reuptake inhibitors (SNRIs), offer a promising approach to addressing menopausal symptoms in women [45]. Extensive research has confirmed the effectiveness of non-hormonal treatments in significantly diminishing the intensity and severity of HF, with reported decreases of up to 70–80% [46]. However, it is crucial to acknowledge that their suitability for women undergoing tamoxifen therapy is subject to specific constraints. More precisely, particular SSRIs and SNRIs, notably paroxetine and fluoxetine, have demonstrated the ability to inhibit the enzyme CYP2D6. This inhibition can lead to a reduction in the levels of the active tamoxifen metabolite, endoxifen [47]. Simultaneously, the consumption of a diet rich in *n*-3 PUFAs has shown the potential to alleviate vasomotor symptoms (VMS), thereby suggesting the possible utility of *n*-3 PUFAs in addressing such symptoms [29]. Within the scope of this study, the trials included demonstrated a heterogeneous nature, with certain trials indicating a decrease in VMS (including HF and night sweats) following *n*-3 PUFAs intervention, while others did not exhibit such effects. For example, Lucas et al. documented a reduction in both frequency and intensity of HF and night sweats in menopausal women who consumed *n*-3 PUFAs capsules [27]. In contrast, Cohen et al. and Reed et al. did not observe substantial effects on VMS through *n*-3 PUFAs treatment. The divergent outcomes might potentially be attributed to variations in dosages, treatment durations, and participant characteristics [28,36].

Sleep disturbances frequently afflict postmenopausal women, often linked to the presence of HF and night sweats [48]. Epidemiologic studies have commonly reported increased sleep disturbances during menopause [49], but laboratory research presents differing insights. One specific study in a laboratory context revealed no notable variations in sleep metrics, performance assessments, or questionnaire responses among premenopausal women, symptomatic postmenopausal women, and asymptomatic postmenopausal women [50]. Perimenopause, a phase characterized by a lack of precise boundaries, encompasses the concluding years of a female’s reproductive cycle with an average of 15–30 pg/mL E2 levels [51,52]. Furthermore, when considering whole-night data, hot flashes were not found to be immediate triggers for awakenings or arousal [50]. Another study involving women aged 44–56 experiencing poor sleep quality highlighted that objective sleep quality primarily correlated with factors such as apneas, periodic limb movements, and arousal, while subjective sleep quality was linked to anxiety levels and the number of hot flashes in the initial half of the night. These findings suggest that anxiety may mediate some reported instances of poor sleep during menopause, underscoring the significance of identifying primary sleep disorders like apnea and periodic limb movements, which can significantly disrupt sleep and hold important medical implications [53]. Several other studies have also revealed a graduated correlation between the frequency and severity of HF and the intensity of insomnia symptoms, accompanied by quantifiable measures of disrupted sleep patterns [54,55]. An intriguing randomized controlled trial displayed noteworthy results; where *n*-3 PUFAs supplementation was employed alongside conventional medication, it improved outcomes spanning depression symptoms, anxiety, sleep dimensions, and emotional self-regulation, surpassing placebo effects [56]. However, our comprehensive systematic analysis did not yield robust evidence supporting the notion that *n*-3 PUFAs supplementation significantly enhances sleep quality in postmenopausal women. In line with this, Guthrie et al., Cohen et al., and Reed et al. all concurred by reporting no substantial impact on sleep quality through diverse sleep assessment scales, including the PSQI and ISI [28,36,41]. Despite the common occurrence of sleep issues in menopausal women, it appears that *n*-3 PUFAs supplementation does not offer discernible efficacy in augmenting sleep quality within this cohort. In contrast, a distinct study highlighted that DHA/EPA supplementation did enhance sleep quality in middle-aged and elderly individuals, even at the lower doses employed in earlier investigations [57]. These disparities in outcomes could potentially be attributed to suboptimal *n*-3 PUFAs doses or an imbalance in the optimal quantities of individual components needed for a comprehensive effect.

Depression, characterized by persistent low mood and reduced interest in daily activities for more than two weeks, is notably more prevalent among females, with 1.5 to 3 times higher incidence rates compared to males [58,59]. A significant predisposing factor for depression during menopause was a prior history of depressive disorder, indicating a recurrence of pre-existing depression. Notably, VMS like insomnia and HF exhibited strong associations with the onset of new depressive episodes, increased anxiety, and the recurrence of pre-existing depressive conditions [60]. It has been postulated that alterations in hormonal profiles throughout the menopausal transition could potentially impact the central nervous system by modulating hypothalamic and hippocampal functions. Steroid hormones can exert an influence on serotonin and gamma-aminobutyric acid (GABA) signaling pathways [61,62]. Additionally, in conjunction with the fluctuations in neuronal opioids observed during menopause, these hormonal changes have been linked to the manifestation of symptoms such as depression, irritability, and anxiety [63]. In seeking relief from depressive symptoms, individuals often turn to antidepressants, particularly SSRIs, despite potential side effects such as sexual dysfunction and weight gain if used over extended periods [64,65]. A recent network meta-analysis (NMA) was conducted to determine the effect of pharmacological interventions and hormone therapies on depressive symptoms in peri and post-menopause women. The NMA presented evidence that fluoxetine plus HRT may be beneficial to menopausal women with a definite diagnosis of depression but not to those without depression, or post-menopausal women [66]. In contrast, emerging research has spotlighted the role of polyunsaturated fatty acids, including *n*-3 PUFAs, in mitigating depressive symptoms [67,68,69]. An intriguing study underscored the clinical efficacy of endocannabinoids derived from *n*-3 PUFAs in the treatment of MDD, opening avenues for innovative therapeutic approaches [70]. However, the impact of *n*-3 PUFAs supplementation on depression among postmenopausal women remains equivocal. Masoumi et al. demonstrated reduced depression scores through combined citalopram and *n*-3 PUFAs supplementation [40]. In contrast, Cohen et al. and Reed et al. did not observe significant changes in depression scores with *n*-3 PUFAs supplementation alone [28,36]. Lucas et al., on the other hand, reported lowered depression scores in women who received *n*-3 PUFAs capsules, suggesting potential benefits in alleviating depressive symptoms [31]. Notably, due to the diverse range of outcomes, prudence is necessary when drawing definitive conclusions about the antidepressant effects of omega-3 supplementation in menopausal women.

VMS, which can significantly compromise women’s quality of life, has often been linked to the menopausal transition. Although prior epidemiological studies have primarily associated this transition with somatic symptoms, the connection to other areas of quality of life remains unclear [71]. A study found that menopausal symptoms significantly reduced the quality of life in affected individuals, leading to decreased productivity and economic implications [72]. Also, a study within this review demonstrated improvements in the MENQOL score among the *n*-3 PUFAs-supplemented group, indicating a potential positive impact on overall well-being during menopause [27]. Additionally, our comprehensive analysis affirms the general safety of *n*-3 PUFAs supplementation in menopausal women, as adverse effects were not prominently noted. This observation aligns with findings from another systematic review conducted to assess the impact of *n*-3 PUFAs supplementation during the menopausal transition [26].

## 5. Strengths and Limitations

Firstly, our systematic review provides a thorough compilation of included studies, all of which were randomized and had a homogenous control group. Secondly, by focusing specifically on postmenopausal women, it addresses a critical demographic group with unique health concerns. Thirdly, its quality assessment enhances the reliability of the conclusions drawn. This systematic review has several important limitations that require attention. Firstly, there were differences in the dosages and frequencies of *n*-3 PUFAs supplementation among the included studies. Secondly, the use of various assessment methods for outcomes introduces the possibility of bias and measurement error. Thirdly, the exclusion of non-English papers may have led to the omission of pertinent data. Lastly, the relatively small sample size and the study’s relatively short duration restrict its ability to capture long-term effects or rare outcomes. These limitations should be considered when interpreting the findings and can serve as guidance for future research efforts aimed at addressing these constraints more thoroughly.

## 6. Conclusions

In summary, the outcomes of our investigation indicate that the impact of *n*-3 PUFA supplementation on menopausal symptoms in postmenopausal women is varied. While certain studies highlight benefits for VMS and mood disturbances, others do not corroborate these effects. The data suggesting a positive influence of *n*-3 PUFA supplementation on sleep quality in menopausal women are limited. Nonetheless, the safety profile of such supplementation remains promising. Therefore, we propose that future research should entail extended follow-up periods, encompass larger cohorts, and explore combined therapeutic approaches with other medications aimed at enhancing the management of menopausal symptoms.

## Figures and Tables

**Table 1:** Study research question (PICOS).

PICOS Components	Determinants
Population (P)	Postmenopausal women
Intervention (I)	*N*-3 PUFAs
Comparison (C)	Control group and placebo
Outcome (O)	Vasomotor Symptoms, Sleep Quality and Depression
Study Design (S)	Randomized Clinical Trials

**Table 2:** The main characteristics of nine included studies evaluating the effect of *n*-3 PUFAs intake on vasomotor symptoms, sleep quality, and depression in postmenopausal women.

Authors & Year	Study Design	Participants, No.	Intervention	Duration	Vasomotor Symptoms	Sleep Quality	Depression	Other Outcomes
[40]	Triple-Blind Randomized Controlled Trial	Menopause women, *n* = 60	Intervention group: 20 mg citalopram and 1 g of *n*-3 PUFAs per day Placebo group: 20 mg citalopram along with a placebo per day	4 weeks	--------	--------	BDI-II (***p* < 0.001**)	------
		Intervention group, *n* = 30 Control group, *n* = 30						
[41]	Double-blind placebo-controlled, RCT	Postmenopausal women, *n* = 188	Intervention group: 1.8 g *n*-3 PUFAs per day Placebo group: 3 capsules per day containing olive oil	12 weeks	--------	PSQ-I (***p* = 0.0933**) ISI (***p*** = 0.729)	------	------
		Intervention group, *n* = 95 Control group, *n* = 93						
[28]	Double-Blind, Randomized Clinical Trial	Menopause women, *n* = 355	Intervention group: 615 mg *n*-3 PUFAs (EPA = 425 mg, DHA = 100 mg) 3 capsules per day Placebo group: 3 capsules per day containing olive oil	12 weeks	VMS frequency (***p*** = 0.283)	PSQ-I (***p*** = 0.0933) ISI (***p*** = 0.729)	PHQ-8 (***p*** = 0.097) GAD-7 (***p*** = 0.191)	No Adverse Effect
		Intervention group, *n* = 177; Placebo group, *n* = 178						
[31]	Double-blind placebo-controlled, RCT	Menopause women, *n* = 120	Intervention group: 500 mg *n*-3 PUFAs (EPA= 350 mg and DHA= 50 mg in ethyl esters form)/day Placebo group: 500 mg capsule containing sunflower oil per day 0.2% of regular fish oil (18% EPA/12% DHA) 3 times daily	8 weeks	-------	-----	PGWB (***p* = 0.034**) HSCL-D-20 (***p* = 0.040**) HAM-D-21 (***p* = 0.030**)	------
		Intervention group, *n* = 59; Placebo group, *n* = 61						
[27]	Double-blind placebo-controlled, RCT	Menopause women, *n* = 120 Intervention group, *n* = 59; Placebo group, *n* = 61	Intervention group: 500 mg *n*-3 PUFAs (EPA = 350 mg and DHA = 50 mg in ethyl esters form)/day Placebo group: 500 mg capsule containing sunflower oil per day 0.2% of regular fish oil (18% EPA/12% DHA) 3 times daily	8 weeks	HF and night sweats Frequency **(*p* = 0.005)** and Intensity (*p* = 0.64)	------	-------	MENQOL (*p* = 0.2) No Adverse Effect
[36]	Randomized control trial	Menopause women, *n* = 355;	Intervention group: *n*-3 PUFAs supplement contained 425 mg ethyl EPA, 100 mg DHA acid per day Placebo group: 90 mg placebo containing olive oil per day	12 weeks	VMS frequency (*p* = 0.06)	PSQ-I (***p*** = 0.0933) ISI (***p*** = 0.729) PSS (***p*** = 0.08)	PHQ-8 (***p*** = 0.097) GAD-7 (***p*** = 0.191)	MENQOL (***p*** = 0.12)
		Intervention group, *n* = 177; Placebo group, *n* = 178						
[37]	Randomized, Prospective, Two-Arm Study	Menopause women, *n* = 76;	Intervention group: *n*-3 PUFAs (425 mg of *n*-3 PUFAs/capsule), 2 capsules per day Placebo group: Soybean isoflavones (54.4 mg of isoflavones/tablet), 2 tablets per day	16 weeks	VMS Frequency and HF **(*p* < 0.001)**	-----	------	No Adverse Effect
		*n*-3 PUFAs group, *n* = 40; Isoflavone group, *n* = 36						
[38]	Double-blind, Placebo-Controlled, Randomized Clinical Trial	Menopause women, *n* = 180;	Intervention group: 1000 mg Omega-rex soft gel Soygan 500 mg capsule Placebo group: placebo	3 months	MRS **(*p* = 0.03)**	-----	-----	No Adverse Effect
		Soy group, *n* = 60; *n*-3 PUFAs group, *n* = 60; Placebo group, *n* = 60						
[39]	Double-blind, randomized controlled clinical trial	Menopause women, *n* = 68;	Intervention group: 300 mg (contain EPA = 120 mg and DHA = 180 mg) per day Placebo group: Placebo containing paraffin	8 weeks	HF frequency **(*p* = 0.003)** but no intensity (*p* = 0.2)	-----	-------	No Adverse Effect
		*n*-3 PUFAs group, *n* = 38; Control, *n* = 38						

Abbreviations: PUFAs, Polyunsaturated Fatty Acids; BDI, Beck’s Depression Inventory; PSQI, Pittsburgh Sleep Quality Index; EPA, Eicosapentaenoic Acid; DHA, Docosahexaenoic acid; VMS, Vasomotor Symptoms; ISI, Insomnia Severity Index; PHQ-8, Physician’s Health Questionnaire depression domains; GAD-7, Generalized Anxiety Disorder questionnaire; PGWB, Psychological General Well-Being Schedule; HSCL-D-20, 20-item Hopkins Symptom Checklist Depression Scale; HAM-D- 21, 21-item Hamilton Depression Rating Scale; HF, Hot flashes; MENQOL, Menopause-specific quality of life score; MRS, Menopause Rating Score.

## Data Availability

Not applicable.

## Supplementary Materials

The following supporting information can be downloaded at: https://www.mdpi.com/article/10.3390/nu15194231/s1, Table S1. PRISMA 2020 Main Checklist; Table S2. Search strategy; Table S3. List of excluded studies.

## Abbreviations

ALA	ALPHA-LINOLENIC ACID
BDI	Beck’s Depression Inventory
CINAHL	Cumulated Index to Nursing and Allied Health Literature
DHA	Deocosahexaenoic acid
EMBASE	Excerpta Medica Database
EPA	Eicosapentaenoic acid
ET	Estrogen Therapy
FDA	Food and Drug administration
GABA	Gamma-Aminobutyric acid
GAD-7	Generalized Anxiety Disorder scale
GNRH	Gonadotropin-releasing hormone
HAM-D-	Hamilton Rating Scale for Depression
HF	Hot flashes
HPA	Hypothalamic-pituitary-adrenal
HRT	Hormone replacement therapy
HSCL-D-20	Hopkins Symptom Checklist Depression Scale
HT	Hormone therapy
ISI	Insomnia Severity Index
IU	International Unit
MDD	Major depressive disorder
MENQOL	Menopause-specific quality of life score
MESH	Medical Subject Headings
MRF4	Muscle regulatory factor 4
MRS	Menopause rating scale
MYOD	Myogenic differentiation factor
NMA	Network meta-analysis
PGWB	Psychological General Well-Being Schedule
PHQ-8	Physician’s Health Questionnaire depression domains
PICO	Population, Intervention, Comparison and Outcome
PICOS	Population, Intervention, Comparison, Outcome and Study
PRISMA	performed according to the systematic reviews and metanalysis
PROSPERO	Preferred Reporting Items for Systematic Reviews and Meta-Analyses.
PSQI	Pittsburgh Sleep Quality Index
PSS	Perceived Stress Score
PUBMED	Player Unknown Battle Mantra Ending In Domination
PUFA	Polyunsaturated fatty acid
RCT	Randomized controlled trial
ROB	Risk of bias assessment
SNRIS	Serotonin-norepinephrine reuptake inhibitors
SSRIS	Selective serotonin reuptake inhibitors
VMS	Vasomotor symptoms
