# Supplementary material for: Effects of Omega-3 Polyunsaturated Fatty Acids Intake on Vasomotor Symptoms, Sleep Quality and Depression in Postmenopausal Women: A Systematic Review
Source: Nutrients. 2023 Sep 30;15(19):4231. doi: 10.3390/nu15194231 (PMC10574492; doi:10.3390/nu15194231)
Supplement: Supplementary file 1 [file nutrients-15-04231-s001.zip › nutrients-2614287-supplementary.pdf]

Table S1. PRISMA 2020 Main Checklist

Topic	No.	Item	Location where item is reported
**TITLE**			
**Title**	1	Identify the report as a systematic review.	1
**ABSTRACT**			
**Abstract**	2	See the PRISMA 2020 for Abstracts checklist	1
**INTRODUCTION**			
**Rationale**	3	Describe the rationale for the review in the context of existing knowledge.	2-3
**Objectives**	4	Provide an explicit statement of the objective(s) or question(s) the review addresses.	2-3
**METHODS**			
**Eligibility criteria**	5	Specify the inclusion and exclusion criteria for the review and how studies were grouped for the syntheses.	4-5
**Information sources**	6	Specify all databases, registers, websites, organisations, reference lists and other sources searched or consulted to identify studies. Specify the date when each source was last searched or consulted.	4 and supplementary data
**Search strategy**	7	Present the full search strategies for all databases, registers and websites, including any filters and limits used.	4 and supplementary data
**Selection process**	8	Specify the methods used to decide whether a study met the inclusion criteria of the review, including how many reviewers screened each record and each report retrieved, whether they worked independently, and if applicable, details of automation tools used in the process.	5 and supplementary data
**Data collection process**	9	Specify the methods used to collect data from reports, including how many reviewers collected data from each report, whether they worked independently, any processes for obtaining or confirming data from study investigators, and if applicable, details of automation tools used in the process.	5

Topic	No.	Item	Location where item is reported
**Data items**	10a	List and define all outcomes for which data were sought. Specify whether all results that were compatible with each outcome domain in each study were sought (e.g. for all measures, time points, analyses), and if not, the methods used to decide which results to collect.	NA
	10b	List and define all other variables for which data were sought (e.g. participant and intervention characteristics, funding sources). Describe any assumptions made about any missing or unclear information.	4-5
**Study risk of bias assessment**	11	Specify the methods used to assess risk of bias in the included studies, including details of the tool(s) used, how many reviewers assessed each study and whether they worked independently, and if applicable, details of automation tools used in the process.	5
**Effect measures**	12	Specify for each outcome the effect measure(s) (e.g. risk ratio, mean difference) used in the synthesis or presentation of results.	NA
**Synthesis methods**	13a	Describe the processes used to decide which studies were eligible for each synthesis (e.g. tabulating the study intervention characteristics and comparing against the planned groups for each synthesis (item 5)).	7-8
	13b	Describe any methods required to prepare the data for presentation or synthesis, such as handling of missing summary statistics, or data conversions.	NA
	13c	Describe any methods used to tabulate or visually display results of individual studies and syntheses.	7-8
	13d	Describe any methods used to synthesize results and provide a rationale for the choice(s). If meta-analysis was performed, describe the model(s), method(s) to identify the presence and extent of statistical heterogeneity, and software package(s) used.	NA
	13e	Describe any methods used to explore possible causes of heterogeneity among study results (e.g. subgroup analysis, meta-regression).	NA

Topic	No.	Item	Location where item is reported
	13f	Describe any sensitivity analyses conducted to assess robustness of the synthesized results.	NA
**Reporting bias assessment**	14	Describe any methods used to assess risk of bias due to missing results in a synthesis (arising from reporting biases).	5
**Certainty assessment**	15	Describe any methods used to assess certainty (or confidence) in the body of evidence for an outcome.	NA
**RESULTS**			
**Study selection**	16a	Describe the results of the search and selection process, from the number of records identified in the search to the number of studies included in the review, ideally using a flow diagram.	5-6 and figure 2
	16b	Cite studies that might appear to meet the inclusion criteria, but which were excluded, and explain why they were excluded.	6 and supplementary data
**Study characteristics**	17	Cite each included study and present its characteristics.	6-9 and Table 2
**Risk of bias in studies**	18	Present assessments of risk of bias for each included study.	9-10
**Results of individual studies**	19	For all outcomes, present, for each study: (a) summary statistics for each group (where appropriate) and (b) an effect estimate and its precision (e.g. confidence/credible interval), ideally using structured tables or plots.	NA
**Results of syntheses**	20a	For each synthesis, briefly summarise the characteristics and risk of bias among contributing studies.	9-10 and figure 3
	20b	Present results of all statistical syntheses conducted. If meta-analysis was done, present for each the summary estimate and its precision (e.g. confidence/credible interval) and measures of statistical heterogeneity. If comparing groups, describe the direction of the effect.	NA
	20c	Present results of all investigations of possible causes of heterogeneity among study results.	NA

Topic	No.	Item	Location where item is reported
	20d	Present results of all sensitivity analyses conducted to assess the robustness of the synthesized results.	NA
**Reporting biases**	21	Present assessments of risk of bias due to missing results (arising from reporting biases) for each synthesis assessed.	9-10
**Certainty of evidence**	22	Present assessments of certainty (or confidence) in the body of evidence for each outcome assessed.	NA
**DISCUSSION**			
**Discussion**	23a	Provide a general interpretation of the results in the context of other evidence.	10-12
	23b	Discuss any limitations of the evidence included in the review.	13-14
	23c	Discuss any limitations of the review processes used.	13-14
	23d	Discuss implications of the results for practice, policy, and future research.	13-14
**OTHER INFORMATION**			
**Registration and protocol**	24a	Provide registration information for the review, including register name and registration number, or state that the review was not registered.	4
	24b	Indicate where the review protocol can be accessed, or state that a protocol was not prepared.	4
	24c	Describe and explain any amendments to information provided at registration or in the protocol.	NA
**Support**	25	Describe sources of financial or non-financial support for the review, and the role of the funders or sponsors in the review.	NA
**Competing interests**	26	Declare any competing interests of review authors.	NA

Topic	No.	Item	Location where item is reported
**Availability of data, code and other materials**	27	Report which of the following are publicly available and where they can be found: template data collection forms; data extracted from included studies; data used for all analyses; analytic code; any other materials used in the review.	Supplementary data

PRISMA Abstract Checklist

Topic	No.	Item	Reported?
**TITLE**			
**Title**	1	Identify the report as a systematic review.	Yes
**BACKGROUND**			
**Objectives**	2	Provide an explicit statement of the main objective(s) or question(s) the review addresses.	Yes
**METHODS**			
**Eligibility criteria**	3	Specify the inclusion and exclusion criteria for the review.	Yes
**Information sources**	4	Specify the information sources (e.g. databases, registers) used to identify studies and the date when each was last searched.	Yes
**Risk of bias**	5	Specify the methods used to assess risk of bias in the included studies.	Yes
**Synthesis of results**	6	Specify the methods used to present and synthesize results.	Yes
**RESULTS**			
**Included studies**	7	Give the total number of included studies and participants and summarise relevant characteristics of studies.	Yes

Topic	No.	Item	Reported?
**Synthesis of results**	8	Present results for main outcomes, preferably indicating the number of included studies and participants for each. If meta-analysis was done, report the summary estimate and confidence/credible interval. If comparing groups, indicate the direction of the effect (i.e. which group is favoured).	Yes
**DISCUSSION**			
**Limitations of evidence**	9	Provide a brief summary of the limitations of the evidence included in the review (e.g. study risk of bias, inconsistency and imprecision).	Yes
**Interpretation**	10	Provide a general interpretation of the results and important implications.	Yes
**OTHER**			
**Funding**	11	Specify the primary source of funding for the review.	No
**Registration**	12	Provide the register name and registration number.	Yes

From: Page MJ, McKenzie JE, Bossuyt PM, Boutron I, Hoffmann TC, Mulrow CD, et al. The PRISMA 2020 statement: an updated guideline for reporting systematic reviews. MetaArXiv. 2020, September 14. DOI: 10.31222/osf.io/v7gm2. For more information, visit: www.prisma-statement.org

Table S2. Search strategy

Database	Search Strategies	Identified studies
Cochrane Library	("omega-3" OR "fish oils" OR "PUFA") AND ("menopause" OR "hot flashes" OR "night sweats" OR "vasomotor") AND ("insomnia" OR "sleep quality" OR "depression") in Title Abstract Keyword -	25
Web of Science	"Omega 3" OR "fish oils" OR "PUFA" (Abstract) and "Menopause" OR "hot flashes" OR "night sweats" OR "Vasomotor" (Abstract) and "insomnia" OR "sleep quality" OR "depression" (Abstract) and Article (Document Types)	10
PubMed	((("omega-3" OR "fish oils" OR "PUFA")) AND (("menopause" OR "hot flashes" OR "night sweats" OR "vasomotor"))) AND (("insomnia" OR "sleep quality" OR "depression"))	27
Embase	('omega-3' OR 'fish oils' OR 'PUFA') AND ('menopause' OR 'hot flashes' OR 'night sweats' OR 'vasomotor') AND ('insomnia' OR 'sleep quality' OR 'depression')	79
CINAHL	("omega-3" OR "fish oils" OR "PUFA") AND ("menopause" OR "hot flashes" OR "night sweats" OR "vasomotor") AND ("insomnia" OR "sleep quality" OR "depression")	18
SCOPUS	("omega-3" OR "fish oil") AND ("menopause" OR "hot flashes" OR "night sweats" OR "vasomotor") AND ("insomnia" OR "sleep quality" OR "depression")	4

Table S3. List of excluded studies

No.	Author	Title	Exclusion
1	Rudin DO	The dominant diseases of modernized societies as omega-3 essential fatty acid deficiency syndrome: Substrate beriberi	Article mismatch
2	Gesteiro E, Megía A, Guadalupe-Grau A, Fernandez-Veledo S, Vendrell J, González-Gross M	Early identification of metabolic syndrome risk: A review of reviews and proposal for defining pre-metabolic syndrome status	Article mismatch
3	Mohammady M, Janani L, Jahanfar S, Mousavi MS	Effect of omega-3 supplements on vasomotor symptoms in menopausal women: A systematic review and meta-analysis	Article mismatch
4	Grigolon RB, Ceolin G, Deng Y, Bambokian A, Koning E, Fabe J, et al	Effects of nutritional interventions on the severity of depressive and anxiety symptoms of women in the menopausal transition and menopause: a systematic review, meta-analysis, and meta-regression	Article mismatch
5	Reed SD, Lacroix AZ, Anderson GL, Ensrud KE, Caan B, Carpenter JS, et al	Lights on MsFLASH: A review of contributions	Article mismatch
6	Crandall CJ, Mehta JM, Manson JE	Management of Menopausal Symptoms: A Review	Article mismatch
7	Wilson MMG	Menopause	Article mismatch
8	Murray MT	MENOPAUSE & ANXIETY	Article mismatch
9	Richardson MK	Menopause Strategies: Finding lasting answers for symptoms and health: Eliminating hot flashes-still not a slam dunk! Menopause	Article mismatch
10	Newton KM, Carpenter JS, Guthrie KA, Anderson GL, Caan B, Cohen LS, et al	Methods for the design of vasomotor symptom trials: The Menopausal Strategies: Finding lasting answers to symptoms and health network	Article mismatch
11	Ciappolino V, Mazzocchi A, Enrico P, Syrén ML, Delvecchio G, Agostoni C, et al	N-3 Polyunsatured Fatty Acids in Menopausal Transition: A Systematic Review of Depressive and Cognitive Disorders with Accompanying Vasomotor Symptoms	Article mismatch
12	Decandia D, Landolfo E, Sacchetti S, Gelfo F, Petrosini L, Cutuli D	n-3 PUFA Improve Emotion and Cognition during Menopause: A Systematic Review	Article mismatch

13	Hall E, Frey BN, Soares CN	Non-hormonal treatment strategies for vasomotor symptoms: A critical review	Article mismatch
14	Brandon AR, Crowley SK, Gordon JL, Girdler SS	Nonpharmacologic Treatments for Depression Related to Reproductive Events	Article mismatch
15	Sánchez-Borrego R, von Schacky C, Osorio MJA, Llaneza P, Pinto X, Losa F, et al	Recommendations of the Spanish Menopause Society on the consumption of omega-3 polyunsaturated fatty acids by postmenopausal women	Article mismatch
16	Albertazzi P	A review of non-hormonal options for the relief of menopausal symptoms	Article mismatch
17	Tester J	Reviews of medical journal articles	Article mismatch
18	Yelland S, Steenson S, Creedon A, Stanner S	The role of diet in managing menopausal symptoms: A narrative review	Article mismatch
19	Landis CA, Woods NF	Seasonality and symptoms	Article mismatch
20	Davies A	A systematic review of the effect of omega-3 long chain polyunsaturated fatty acids on menopausal symptoms (vasomotor, depression and insomnia) of women going through the menopause transition	Article mismatch
21	Grigoriadis S, Kennedy S, Robinson G, VonderPorten E, Mamisashvili L, Peer M	A systematic review of treatments for depression in perimenopausal and postmenopausal women	Article mismatch
22	Tal JZ, Suh SA, Dowdle CL, Nowakowski S	Treatment of insomnia, insomnia symptoms, and obstructive sleep apnea during and after menopause: Therapeutic approaches	Article mismatch
23	Saldeen P, Saldeen T	Women and omega-3 Fatty acids	Article mismatch
24	BMS Annual Scientific Conference 2022	Post Reproductive Health	Conference paper
25	Reed SD, La Croix AZ, Guthrie KA, Anderson G, Ensrud K, Caan B, et al	A Decade of MsFLASH Findings - Time to Get the Word Out	Conference paper
26	Masoumi SZ	Compersssion with the combined effects of omega _3 and citalopram for depression during menopause in postmenopausal women referred to health centers in Hamadan city in 1391: a double-blind clinical trial	duplicate
27	Irct2013052113405N	Effect of omega3 in depression in menopause	duplicate
28	Irct2017011131880N	The Comparison of the Effect of Omega3 and Folic Acid Oral Tablet on Hot Flashes in Menopausal Women	Full text not available
29	Tadayon Najaf Abadi M, Shalikar Z, Abedi P	The impact of Omga-3 fatty acids on depression of post menopausal women	Full text not available

30	Nct	Omega-3 for Peri- and Postmenopausal Depression	Full text not available
31	Li D, Wu Q, Xu W, Zheng H, Tong Y, Li Y	Dietary manganese intake is inversely associated with depressive symptoms in midlife women: A cross-sectional study	Irrelevant Intervention
32	Verde L, Barrea L, Vetrani C, Frias-Toral E, Chapela SP, Jayawardena R, et al	Chronotype and Sleep Quality in Obesity: How Do They Change After Menopause? Current Obesity Reports	No relevant outcome data
33	Deligiannidis KM, Freeman MP	Complementary and Alternative Medicine for the Treatment of Depressive Disorders in Women	No relevant outcome data
34	Özcan H, Çakmak S, Salman E	Complementary and alternative medicine methods used for sleep disturbance in menopause	No relevant outcome data
35	Lucas M, Dodin S, Poulin MJ, Merette C	Does omega-3 improve menopausal symptoms? Agro Food Industry Hi-Tech	No relevant outcome data
36	Irct2012071410281N	Effect of Omega 3 supplementation in women with polycystic ovary syndrome	No relevant outcome data
37	Irct201112318564N	Effect of omega3 supplementation in women with polycystic ovary syndrome	No relevant outcome data
38	Diem SJ, LaCroix AZ, Reed SD, Larson JC, Newton KM, Ensrud KE, et al	Effects of pharmacologic and nonpharmacologic interventions on menopause-related quality of life: a pooled analysis of individual participant data from four MsFLASH trials	No relevant outcome data
39	Newton KM, Reed SD, Guthrie KA, Sherman KJ, Booth-LaForce C, Caan B, et al	Efficacy of yoga for vasomotor symptoms: a randomized controlled trial	No relevant outcome data
40	Carpenter JS, Tisdale JE, Larson JC, Sheng Y, Chen CX, Von Ah D, et al	MsFLASH analysis of diurnal salivary cortisol and palpitations in peri- and postmenopausal women	No relevant outcome data
41	LaCroix AZ, Joffe H, Cohen LS, Newton KM, Reed SD, Guthie KA, et al	The MsFLASH research network: Latest findings	No relevant outcome data
42	García-Montero C, Ortega MA, Alvarez-Mon MA, Fraile-Martinez O, Romero-Bazán A, Lahera G, et al	The Problem of Malnutrition Associated with Major Depressive Disorder from a Sex-Gender Perspective	No relevant outcome data
43	Shikh EV, Makhova AA	Protective effects of omega-3 polyunsaturated fatty acids in female health	No relevant outcome data
44	Okereke OI	Vitamin D and Omega-3 Fatty Acids: Do They Have Benefits for Mood, Depression, or Cognition? Menopause	No relevant outcome data

45	Von Hagens C, Reinhard-Hennch B, Strowitzki T	Alternative therapies for menopausal women	Not English
46	Chae M, Park K	Association between dietary omega-3 fatty acid intake and depression in postmenopausal women	Not RCT
47	Terauchi M, Odai T, Hirose A, Kato K, Miyasaka N	Chilliness in Japanese middle-aged women is associated with anxiety and low n-3 fatty acid intake	Not RCT
48	Terauchi M, Odai T, Hirose A, Kato K, Akiyoshi M, Miyasaka N	Chilliness in Japanese middle-aged women is associated with low intake of n-3 fatty acids: a cross-sectional study	Not RCT
49	Otte JL, Rand KL, Landis CA, Paudel ML, Newton KM, Woods N, et al	Confirmatory factor analysis of the Pittsburgh Sleep Quality Index in women with hot flashes	Not RCT
50	Abshirini M, Siassi F, Koohdani F, Qorbani M, Khosravi S, Aslani Z, et al	Higher intake of dietary n-3 PUFA and lower MUFA are associated with fewer menopausal symptoms	Not RCT
51	Carpenter J, Woods NF, Otte J, Guthrie K, Hohensee C, Newton KM, et al	MsFLASH participants' priorities for alleviating menopausal symptoms	Not RCT
52	Freeman MP, Hibbeln JR, Silver M, Hirschberg AM, Wang B, Yule AM, et al	Omega-3 fatty acids for major depressive disorder associated with the menopausal transition: a preliminary open trial	Not RCT
53	Oldra CM, Benvegnú DM, Silva DRP, Wendt GW, Vieira AP	Relationships between depression and food intake in climacteric women	Not RCT
54	Shon J, Seong Y, Choi Y, Kim Y, Cho MS, Ha E, et al	Meal-Based Intervention on Health Promotion in Middle-Aged Women: A Pilot Study	Pilot study
55	Jin Y, Kim TH, Park Y	Association between erythrocyte levels of n-3 polyunsaturated fatty acids and depression in postmenopausal women using or not using hormone therapy	Subject irrelevant
56	Da Rocha RVO, Martins MIM, Antunes FTT, Martins MG, Klein AB, Corrêa DS, et al	Behavioral, Oxidative, and Biochemical Effects of Omega-3 on an Ovariectomized Rat Model of Menopause	Subject irrelevant
57	Irct201108237405N	Comparison of the therapeotic effects of omega3 fatty acids and Rose damascena on premenstural syndrom	Subject irrelevant
58	Barnes C, Mitchell P	Considerations in the management of bipolar disorder in women	Subject irrelevant
59	Khalili M, Karimi H, Sadri S	Effect of fish oil supplement and weight loss diet on depression in obese women	Subject irrelevant

60	Tsuboi H, Sakakibara H, Matsunaga M, Tatsumi A, Yamakawa-Kobayashi K, Yoshida N, et al	Omega-3 eicosapentaenoic acid is related to happiness and a sense of fulfillment—a study among female nursing workers	Subject irrelevant
61	Rhee EJ, Kim HC, Kim JH, Lee EY, Kim BJ, Kim EM, et al	2018 Guidelines for the Management of Dyslipidemia in Korea	Unrelated
62	Lim PHC	ABC's of managing andropause & somatopause for the urologist	Unrelated
63	Turner L	The Best Diet for Menopause	Unrelated
64	Barton DL, Loprinzi C, Jatoi A, Vincent A, Limburg P, Bauer B, et al	Can complementary and alternative medicine clinical cancer research be successfully accomplished? The Mayo Clinic-North Central Cancer Treatment Group experience	Unrelated
65	MacQueen GM, Frey BN, Ismail Z, Jaworska N, Steiner M, Lieshout RJV, et al	Canadian Network for Mood and Anxiety Treatments (CANMAT) 2016 clinical guidelines for the management of adults with major depressive disorder: Section 6	Unrelated
66	Mehta PK, Wenger NK	Coronary heart disease in women: Battle is won, but the war remains	Unrelated
67	Prakash S	Current perspective of growth differentiation factor- 15 in changing of lifestyle diseases: An approach for translational research	Unrelated
68	Raič M	Depression and Heart Diseases: Leading Health Problems	Unrelated
69	Whang W, Kubzansky LD, Kawachi I, Rexrode KM, Kroenke CH, Glynn RJ, et al	Depression and Risk of Sudden Cardiac Death and Coronary Heart Disease in Women	Unrelated
70	Colangelo LA, Ouyang P, Golden SH, Szklo M, Gapstur SM, Vaidya D, et al	Do sex hormones or hormone therapy modify the relation of n-3 fatty acids with incident depressive symptoms in postmenopausal women? The MESA Study	Unrelated
71	Markland A, Vaughan C, Huang A, Tangpricha V, Kim E, Bubes V, et al	Effect of vitamin d supplementation on urinary incontinence in post-menopausal women	Unrelated
72	Irct2014020316465N	Evaluating the effects of melatonin on weight and depression	Unrelated
73	Mosca L, Appel LJ, Benjamin EJ, Berra K, Chandra-Strobos N, Fabunmi RP, et al	Evidence-based guidelines for cardiovascular disease prevention in women	Unrelated
74	Silva T, Jesus M, Cagigal C, Silva C	Food with influence in the sexual and reproductive health	Unrelated
75	Fernandes CE, Neto JSDLP, Gebara OCE, De Andrade JP,	I Brazilian guidelines for prevention of cardiovascular disease and influence of	Unrelated

	Neto AMP, Pereira Filho AS, et al	hormone replacement therapy in climacteric women	
76	Journal watch	World of Irish Nursing & Midwifery	Unrelated
77	Condorelli R, Vaz-Luis I	Managing side effects in adjuvant endocrine therapy for breast cancer	Unrelated
78	Perez-Lopez F	The Mediterranean lifestyle and longevity	Unrelated
79	Fouad S, El Shebini SM, Abdel-Moaty M, Ahmed NH, Hussein AMS, Essa HA, et al	Menopause anxiety and depression; how food can help? Open Access Macedonian Journal of Medical Sciences	Unrelated
80	Dickson GM	Menopause management: How you can do better	Unrelated
81	La Croix AZ, Freeman EW, Ensrud K, Reed SD	New clinically relevant findings from the msflash research network: The escitalopram trial	Unrelated
82	Chedraui P, Pérez-López FR	Nutrition and health during mid-life: searching for solutions and meeting challenges for the aging population	Unrelated
83	Daugherty KK, Subramanian J	Pravastatin-induced hepatotoxicity in a patient with fatty liver disease: A case presentation with literature review	Unrelated
84	Irct20191127045525N	Probiotic applications in obesity and related mood disorders	Unrelated
85	Nasrallah HA	Psychopharmacology throughout the life cycle of women	Unrelated
86	Prakash S	Role of antioxidants in changing of lifestyle diseases: A frontier for translational research	Unrelated
87	Hagey AR, Warren MP	Role of exercise and nutrition in menopause	Unrelated
88	Galletly C, Castle D, Dark F, Humberstone V, Jablensky A, Killackey E, et al	Royal Australian and New Zealand College of Psychiatrists clinical practice guidelines for the management of schizophrenia and related disorders	Unrelated
89	Tctr	Safety evaluation and effects of consumption of functional drink containing tuna oil on working memory, emotional stresses, and aging process in perimenopausal and menopausal women	Unrelated
90	Seralthan K, Baskaran H	A short voyage towards flaxseed	Unrelated
91		Some non-hormonal therapies help control menopause symptoms, but they are not as effective as estrogen replacement therapy	Unrelated
92	Tomillero A, Moral MA	Summary	Unrelated
93	Pines A	Three alternative ways to treat VMS failed	Unrelated

94		A variety of non-hormonal strategies may provide women with relief from vasomotor symptoms	Unrelated
95	Manson J	Vitamin D and calcium in midlife women	Unrelated
96	Manson JAE	Vitamin D and cancer and cardiovascular disease: Ready for prime time? Menopause	Unrelated
97	Stewart M	Why are we Told Only Half the Story about HRT and Dementia? Positive Health	Unrelated
98	Wu B, Song Q, Zhang Y, Wang C, Yang M, Zhang J, et al	Antidepressant activity of ω-3 polyunsaturated fatty acids in ovariectomized rats: role of neuroinflammation and microglial polarization	Unsuitable population
99	Dornellas APS, Boldarine VT, Pedroso AP, Carvalho LOT, de andrade IS, Vulcani-Freitas TM, et al	High-Fat Feeding Improves Anxiety-Type Behavior Induced by Ovariectomy in Rats	Unsuitable population
100	Liu XH, Hao JH, Yao ES, Cao J, Zheng XL, Yao D, et al	Polyunsaturated fatty acid supplement alleviates depression-incident cognitive dysfunction by protecting the cerebrovascular and glymphatic systems	Unsuitable population
